# Digital conversations about depression among Hispanics and non-Hispanics in the US: a big‐data, machine learning analysis identifies specific characteristics of depression narratives in Hispanics

**DOI:** 10.1186/s12991-021-00372-0

**Published:** 2021-11-29

**Authors:** Ruby Castilla-Puentes, Anjali Dagar, Dinorah Villanueva, Laura Jimenez-Parrado, Liliana Gil Valleta, Tatiana Falcone

**Affiliations:** 1 Center for Clinical and Translational Science and Training, University of Cincinnati Academic Health Center, Cincinnati, OH USA; 2 Neuroscience- Janssen Research & Development, LLC, Titusville, NJ USA; 3 Hispanic Organization of Leadership and Achievement, HOLA, Employee Resource Group of Johnson & Johnson, New Brunswick, NJ USA; 4 Department of Psychiatry/Epilepsy, Cleveland Clinic Lerner College of Medicine, Cleveland, OH USA; 5 Investigation Group - Sleep Disorders and Forensic Psychiatry, Universidad Nacional de Colombia, Bogota, Colombia; 6 CulturIntel Inc, New York, NY USA

**Keywords:** Depression, Hispanics, Artificial intelligence, Latinos, Big data, Machine learning

## Abstract

**Background:**

Digital conversations can offer unique information into the attitudes of Hispanics with depression outside of formal clinical settings and help generate useful information for medical treatment planning. Our study aimed to explore the big data from open‐source digital conversations among Hispanics with regard to depression, specifically attitudes toward depression comparing Hispanics and non-Hispanics using machine learning technology.

**Methods:**

Advanced machine‐learning empowered methodology was used to mine and structure open‐source digital conversations of self‐identifying Hispanics and non-Hispanics who endorsed suffering from depression and engaged in conversation about their tone, topics, and attitude towards depression. The search was limited to 12 months originating from US internet protocol (IP) addresses. In this cross-sectional study, only unique posts were included in the analysis and were primarily analyzed for their tone, topic, and attitude towards depression between the two groups using descriptive statistical tools.

**Results:**

A total of 441,000 unique conversations about depression, including 43,000 (9.8%) for Hispanics, were posted. Source analysis revealed that 48% of conversations originated from topical sites compared to 16% on social media. Several critical differences were noted between Hispanics and non-Hispanics. In a higher percentage of Hispanics, their conversations portray “negative tone” due to depression (66% vs 39% non-Hispanics), show a resigned/hopeless attitude (44% vs. 30%) and were about ‘living with’ depression (44% vs. 25%). There were important differences in the author's determined sentiments behind the conversations among Hispanics and non-Hispanics.

**Conclusion:**

In this first of its kind big data analysis of nearly a half‐million digital conversations about depression using machine learning, we found that Hispanics engage in an online conversation about negative, resigned, and hopeless attitude towards depression more often than non-Hispanic.

**Supplementary Information:**

The online version contains supplementary material available at 10.1186/s12991-021-00372-0.

## Introduction

The size and diversity of the Hispanic/Latino population in the United States (US) have dramatically increased in the last decade. By 2060, the number of Hispanics in the US is projected to grow to 129 million (31%) [1]. Access to care continues to be a major public health challenge for Hispanic/Latinos [2, 3], and depression is frequently misdiagnosed and often remains untreated among them [4]. Identifying barriers to access depression treatment among Hispanics/Latinos is a critical task. These barriers could be related to an individual, cultural, or healthcare system [5]. Such barriers are more common in communities with strong ethnic identity and recent migrants [6] Additionally, Hispanic/Latino youth avoid formal health care and more often use home remedies and traditional healers [7].

Studies show that depression increases in Hispanic adults as they acculturate in the US [8]. Additionally, there is under-recognition of depression among them, which is multifactorial, including language barrier, and lower health literacy [9]. There is cultural stigma associated with mental illness and depression in the Hispanic population [10, 11]. It might be an important barrier, which prevents an individual from sharing feelings and concerns about depression with immediate family and friends. However, the availability of online forums (e.g. Beyond Blue), topical sites (e.g. Depression and Bipolar Support Alliance), and social media (e.g. Facebook) may help them share and discuss some of these feelings. Therefore, the analysis of these online digital conversations can provide us with valuable information that we would not have otherwise using the sources based on traditional clinical research data.

Although studies have explored the use of technology and social media to help Hispanics suffering from depression [12, 13], prior research has not analyzed the digital conversations of Hispanics suffering from depression at a large scale. According to a recent Pew research center study, nearly 70% of Hispanics are avid users of at least one social media site [14]. People are more likely to share their truthful, uninhibited beliefs and sentiments on social media than with their mental health provider. Therefore, the analysis of such digital conversations provides a unique opportunity to understand the patient’s real-world concerns and mindsets. There is a lot of growing interest in accessing the untapped opportunities for disease surveillance using social media [15]. Digital conversations have helped analyze the barriers to breast cancer treatment [16] and understand the perceptions about suicidality among adolescents and adults with epilepsy [17]. The digital conversations are extracted using computer tools that can extract, i.e. mine, a large amount of data from various internet website, which can then be analyzed by artificial intelligence tools. The use of artificial intelligence (AI; i.e. non-human, computer-based intelligence) in daily life is ubiquitous and its use in healthcare is becoming routine in fields like Radiology and Pathology due to its major strengths in pattern recognition. However, in recent years, AI-based tools have been used in psychiatry research as well [18]. Natural language processing (NLP) is a subfield of AI that helps computers to process and analyze large amounts of natural human language text data, leading to its segmentation based on the conversation topics. NLP has shown promise in detecting depression [19, 20] as well as suicidal ideation and suicidal attempts from review of clinical records [21].

Of note, most national government agencies, such as the U.S. Census Bureau, and research organizations, have opted to use Hispanics and Latinos interchangeably [22]. We are mindful that members of this population may choose to self-identify as one or the other group. However, for reader’s clarity, we chose to use ‘Hispanics’ in the manuscript to represent both the abovementioned groups.Overall, our study aims to use the open-source digital conversations across the entire digital footprint to understand the mindset and sentiments about depression among Hispanics compared to the non-Hispanic population.

## Methods

All the information gathered from the different online, open sources [topical sites (e.g. Depression and Bipolar Support Alliance), message boards (e.g. Beyond Blue), social networks (e.g. Facebook), and blogs] is in the public domain and is de-identified. The study was exempt from Institutional Review Board approval as it used publicly available, de-identified information.

### Search strategy, data extraction, and collection

The populations of interest for this study were Hispanics and non-Hispanics (based on their self-identification during digital conversations or on their public online profile), who endorsed suffering from depression or engaged in conversation about depression. CulturIntel™, an information technology company that analyzes every available open-source digital discussion to discover patterns in the unsolicited digital conversation of the people, used its advanced AI-based tools to mine and structure the unstructured, qualitative online data on the topic of interest (depression). The search was limited to 12-month period, ending on January 1, 2019. The search was limited to English conversations originating from USA internet protocol (IP) addresses.

CulturIntel™ big data and AI suite of tools ‘scrapes and listens’ to open-source conversations online. The data mining and collection occurs across various sites (topical sites, blogs, social network, and message boards) where relevant discussions are taking place. It encompasses a complete range of social discussion channels, including sites directed toward selected segments and directed by (but not limited to) their predefined topic of interest (Hispanics and depression). Advanced search techniques of web crawlers/scrappers (Google technology that archives (copies and stores) a pre-programmed collection of website/topic/discussion data, as it generated on the internet) were applied. CulturIntel™ then extracted the topical data, tagged these data with their origin and user, which were then de-identified. Subsequently a large, unstructured ‘big’ dataset is created. After the completion of this comprehensive dataset, NLP, and text analytics were employed to examine previously described and undescribed patterns in the data. NLP analyses were supervised, whereby the authors worked with CulturIntel™ team in analyzing and tagging a sub-set of the ‘big’ dataset. In this process, digital conversations were reviewed by authors and based on understanding of English language; each conversation was tagged for the positive or negative tone, attitude, etc. towards depression. This random sub-set was used for initial training, testing, and reviewing of a larger data-set by NLP tools, followed by multiple iterations and repetitions of the process leading to supervised machine learning. In this thematic analysis, authors tagged and sorted the data, determined key motivations of topics being discussed, and assigned underlying drivers, attitudes, and topics.

### Data analysis

To avoid duplicity of posts/conversation, multiple postings by an individual were included in the analysis, only if their posts were unique comments. A single user with multiple posts/comments within a conversation was only counted once. A single comment repeatedly appearing through sharing/linking was counted and analyzed only once as well. However, users posting multiple unique comments across discussions/posts/sites were counted for each comment. This was a cross-sectional study and all the conversations from the entire study period were included in the final analysis. We did not divide the study period into smaller epochs to compare changes in digital conversation trends over time. Additionally, the anonymized nature of digital conversations precludes analyzing changes in conversation from individuals during the study period.

The digital conversations were primarily analyzed for their tone, topic, and attitude towards depression using supervised NLP, as described above. This was achieved through two separate frameworks. First, a broader classification system analyzed the conversations about depression based on their tone—positive, neutral, or negative. Second, we further analyzed the conversation with positive and negative tones to group them into heuristic drivers (leading factors) behind the tone, as defined in Table 1.

**Table 1:** Description of negative and positive drivers influencing the tone of digital conversations

Tone	Factor	Description	Hispanics (%)	Non-Hispanics (%)
Negative	Life impact	Negative impact of depression in their life	18	26
	Toll on others	Negative impact of their state of depression on other people close to them	21	19
	Treatment complications	Negative impact of the treatment on their life	26	20
	Stigma	Fear of being labeled as “a crazy person”	21	12
	Lack of treatment efficacy & others	Fact that the treatment is not showing the desired result	14*	23
Positive	Support	Support they have received from their HCP, their caregiver, friends and others	25	22
	Treatment efficacy	Perceived positive impact of the treatment in their lives	19	28
	Enablement/control	A new found control over the condition, which enables them to get back to their lives	25	26
	Enhanced outlook	More positive perspective on their life, usually expressed when their state of depression is under control and treated	28	24
	Others	Other motivations to be positive when talking about depression	3	0

*Includes 5% conversations that could not be classified into the described negative factors and were labelled ‘others’

It is understood that the conversations may differ among individuals based on their status in the natural course and self-management of depression. Therefore, the topics and attitudes were analyzed in a framework that mapped the digital conversations into four possible stages during the journey of depression: (1) Suspect (concerned about the possibility of depression), (2) diagnosis, (3) treating (undergoing active treatment changes), (4) coping (just enduring depression, and lacking constructive perspective to manage it). Of note, not all subjects may go through all these stages. Additionally, due to the cross-sectional study design, the conversations do not represent an individual’s digital conversation spanning several years, through each stage. We performed descriptive statistical analysis whereby categorical variables were analyzed as frequency or percentages.

## Results

**Fig. 1:**
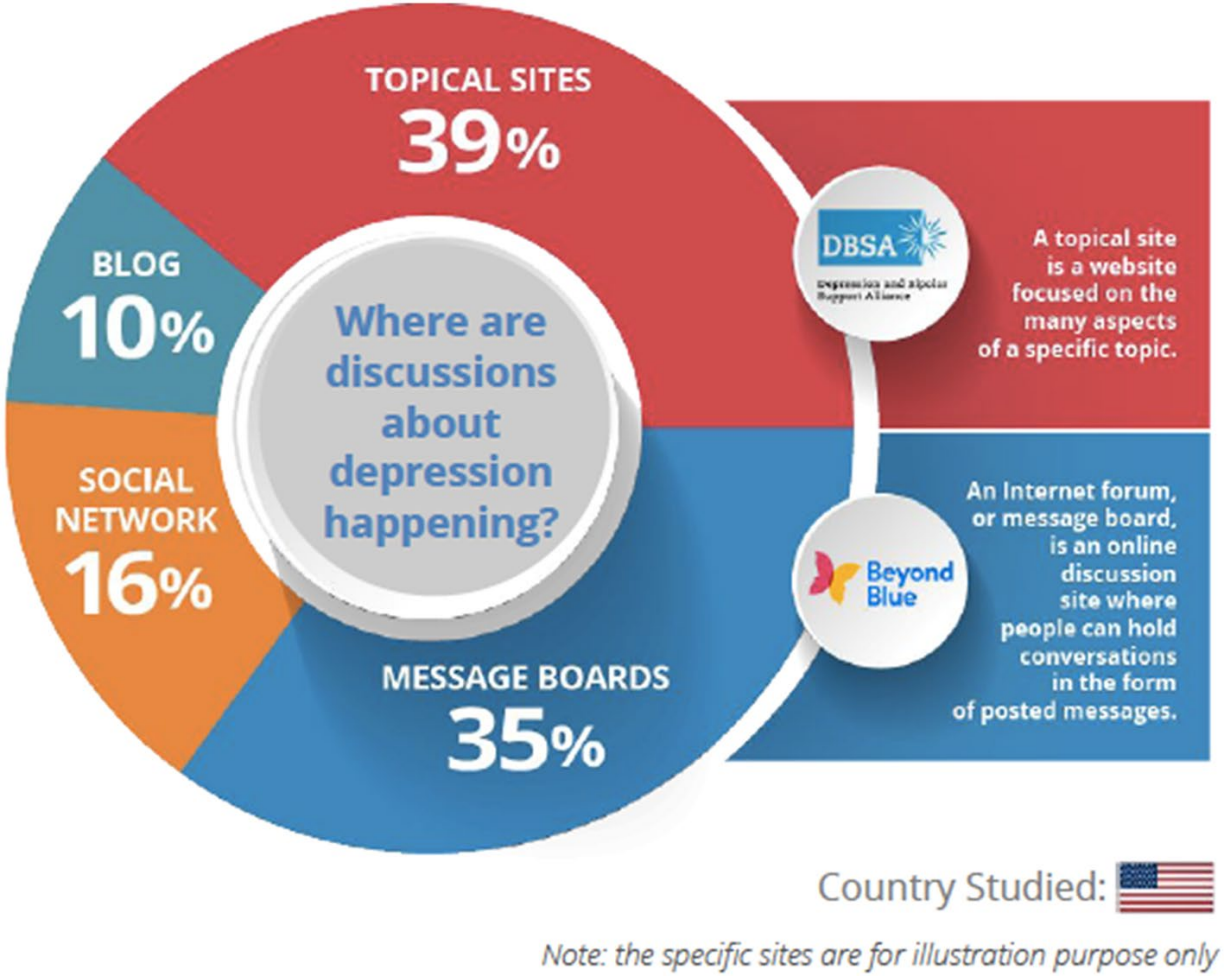
Origins of posts on depression by Hispanics

A total of 441,000 unique open-source conversations about depression were posted online during the study period among the self-identified Hispanics and non-Hispanics. A total of 43,000 (9.8%) conversations were posted online by Hispanics and 398,000 (90.2%) by non-Hispanics. Source analysis revealed that 211,770 (48.0%) conversations originated from topical sites (e.g., Depression and Bipolar Support Alliance—https://www.dbsalliance.org) compared to 70,560 (16%) on social media. Figure 1 provides a complete breakdown of the source of these conversations.

### Tone and their drivers

The content analysis of the conversations by Hispanics shows that 66% of their conversations portray negative tone as compared to 39% among non-Hispanics. The drivers of negative emotions and of positive tone in the conversations were grouped into five most important factors, based on their frequency. Table 1 details these underlying factors that shape the conversation about depression among Hispanics and non-Hispanics.

### Journey through depression

The mapping of digital conversations shows four possible stages (suspect, diagnosis, treating, coping, as described above and examples given in (Additional file 1: Table S1) that a patient journeying through depression undergoes. This analysis shows that the conversations by Hispanics reflect the individual being in a coping phase of depression 1.8 times more often than non-Hispanics (46% vs. 25%, respectively). On the contrary, they are only 0.6 times in the treatment stage of depression compared to non-Hispanics (21% vs. 38%, respectively). A comparable percentage of Hispanics and non-Hispanics were in the suspect (22% each) or diagnosed (11% vs. 15%) stage of depression.

### Attitude towards depression

**Fig. 2:**
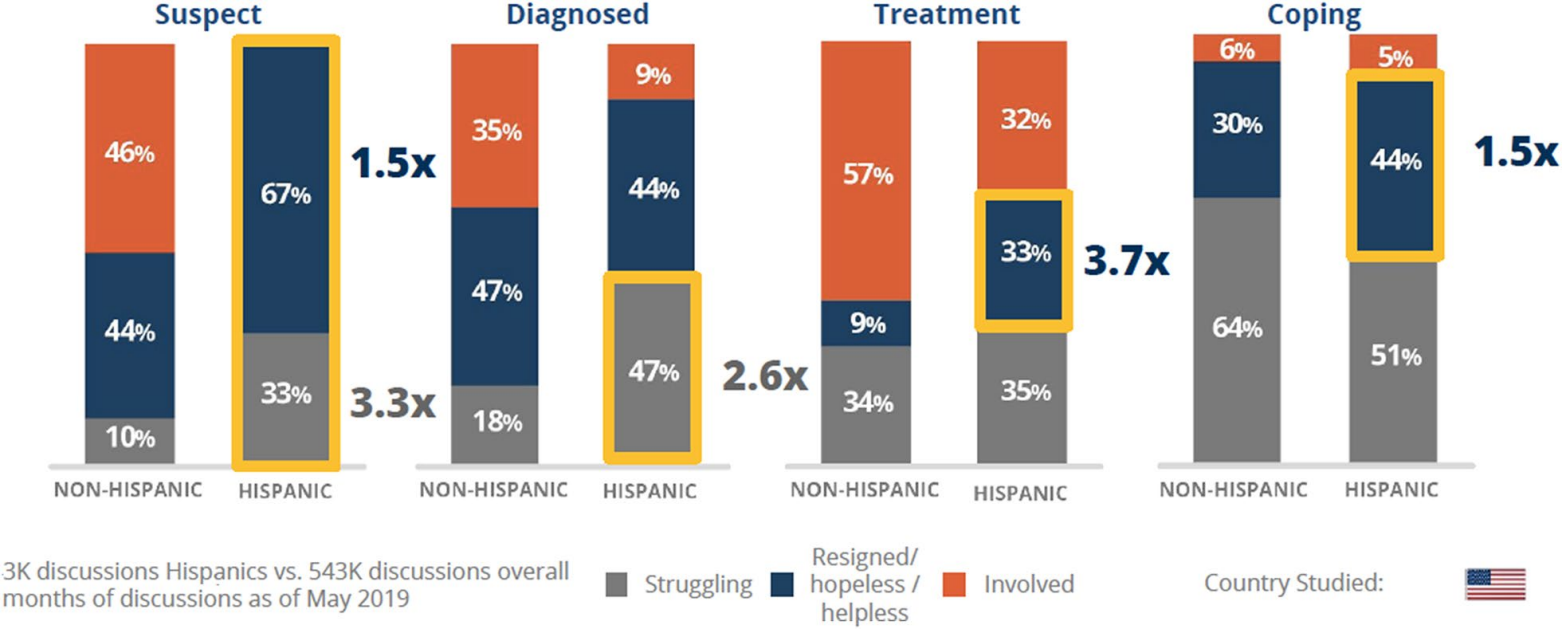
Attitudes while going through depression

The attitudes towards depression were classified into (1) struggling (trying to deal with the situation), (2) resigned/hopeless (aware and accepting their condition as inevitable), and (3) involved (Additional file 1: Table S1). As noted in Fig. 2, a substantially higher percentage of Hispanics have a resigned/hopeless attitude in every stage of depression, except diagnosis, compared to non-Hispanics. They do not exhibit an involved attitude during the suspect stage and come into the diagnosis stage with a struggling attitude, which is 2.6 times more common than non-Hispanics. The disparity in attitude remains in the treatment stage as well, during which one-third Hispanics remain resigned/hopeless, which is 3.7 times as much compared to non-Hispanics. In the coping stage, Hispanic and non-Hispanic are comparable in having an involved attitude, but the conversation among the former group remains more frequently reflect a resigned/hopeless attitude (44% vs. 30%).

### Topic analysis

**Fig. 3:**
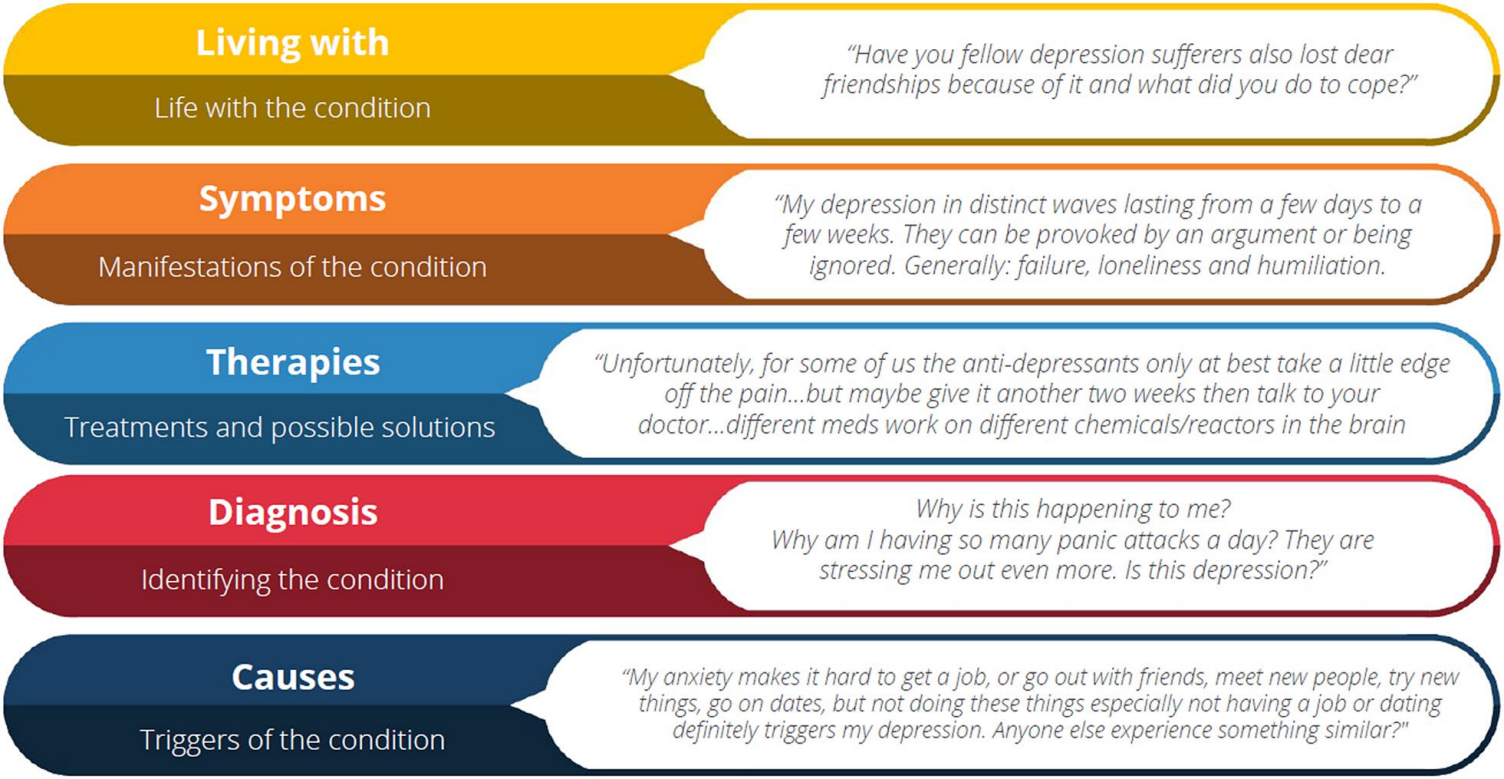
Topics grouping of digital conversations

**Fig. 4:**
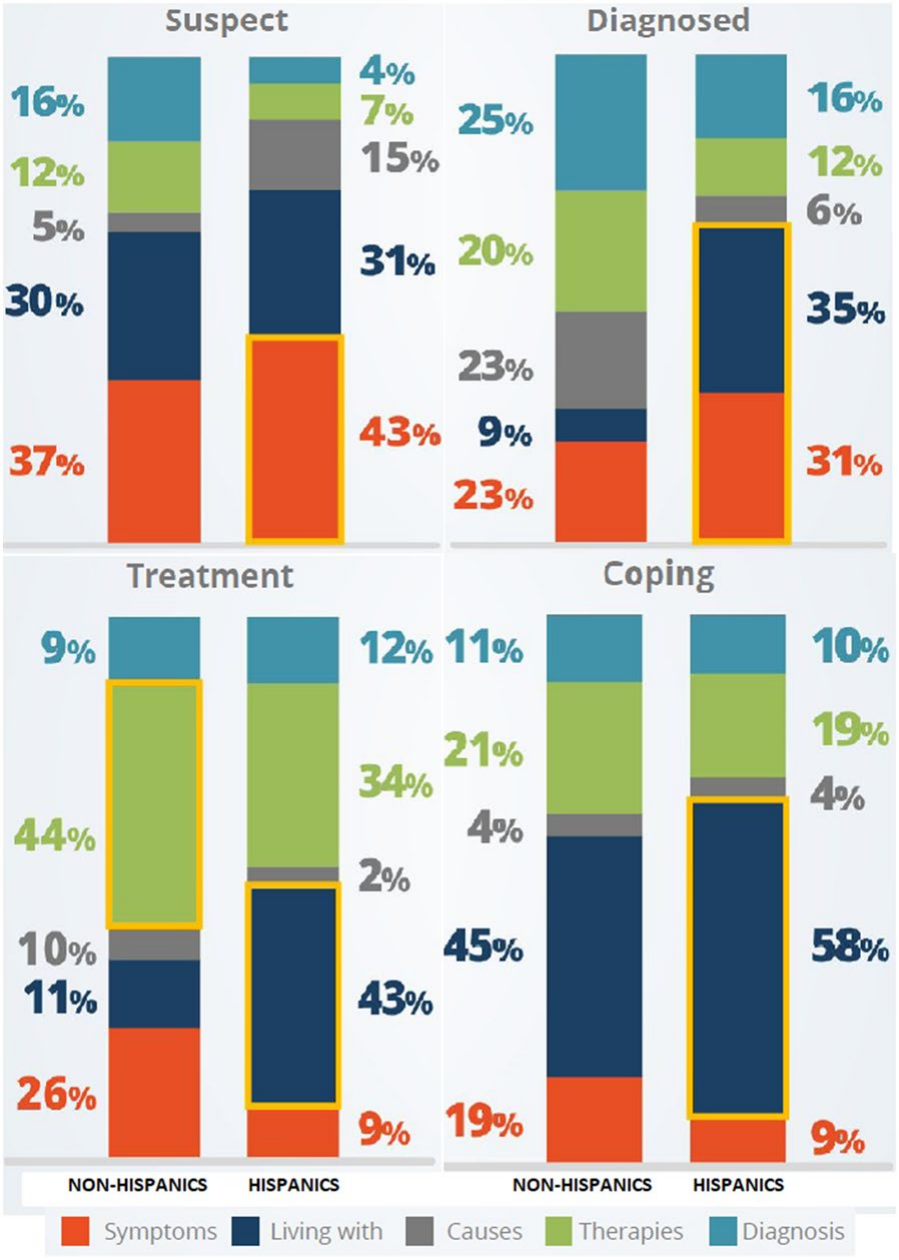
Distribution of topics of conversation among non-Hispanics and Hispanics based on various stages of depression

The conversations were categorized into five topics: living with (talking about life with the condition), symptoms (discussing the manifestations of the condition), therapies (talking about treatment and possible solutions), diagnosis (conversations about identifying the condition), and causes (discussing the triggers of the condition). Figure 3 shows these five topics with a representative digital conversation. The percentage of digital conversation on the topic of symptoms and diagnosis of depression among Hispanics and non-Hispanics were comparable (26% vs. 23%, 12% vs. 16%, respectively). Half as many conversations among Hispanics were about the causes and therapies for depression compared to non-Hispanics (6% vs. 11%, 12% vs. 25%, respectively). Twice as many conversations among Hispanics compared to non-Hispanics were about ‘living with’ depression (44% vs. 25%). Figure 4 shows the distribution of digital conversation topics based on the stage of depression among Hispanics and non-Hispanics.

## Discussion

Using close to half a million unique online digital conversations about depression, analyzed through machine learning techniques like NLP, our study shows a remarkable difference in the attitudes, beliefs, and treatment-seeking behavior towards depression between Hispanics and non-Hispanics. Depression does not discriminate based on race, color, gender or ethnicity. However, the experiences and how people understand and cope with these conditions may be impacted by their cultural beliefs. In Hispanics, protective factors like individual resilience and the family role have been proposed [23, 24]. However, our study reveals a starkly different and dismal picture of how Hispanics cope and approach depression compared to the non-Hispanic population. The analysis of digital conversations found that Hispanic’s discussions about depression more often have a negative tone. Their attitude suggests frequent struggle and a sense of hopelessness about depression. To compound the problem, they are less involved with the treatment and infrequently discuss it online.

The use of digital and social media to research various health metrics adds a new dimension to enhance our understanding of disease perception in the public. These platforms provide ground for collaborative interaction between individuals of diverse backgrounds and health literacy [25]. While some studies find the use of social media to be associated with increased risk of depression [26], others have found its use in disseminating positive health messages about mental health [27]. A recent study found that the analysis of digital comments of an online community on individual posts, in combination, could predict depression in the individual online users [28]. We found that a significantly greater percentage of adolescents compared to adults engage in conversation about suicide. There are several key differences among this sub-population of individuals regarding suicidality, which were revealed by the analysis of digital conversations [29].

Interestingly, only 16% of the digital conversations analyzed were from social networking sites compared to slightly over a third coming from topical sites and message boards. Although the possibility that digital conversations on social media sites are less often open source cannot be ruled out, this distribution of sampled conversations could also reflect a hesitation and reluctance about discussing one’s mental health status, especially depression with a social group of friends and family [29].

The comparison of the digital conversations about depression reveals that 1.7 times as many Hispanics discuss it in negative tones as compared to non-Hispanics. The evaluation of drivers behind the tone of the conversation explains some of this difference. While Hispanics are less concerned about the impact of depression on their lives than non-Hispanics (18% vs. 26%, respectively), a disproportionately higher number of them seem to be influenced by the stigma of depression (21% vs. 12%, respectively). Research reveals a high degree of stigma towards depression in the Hispanic population, which is associated with poor compliance with treatment [30, 31]. While the digital conversation analysis does not assess medication non-compliance or non-adherence of the users, which is known to be higher among Hispanics [32], we found a notable difference in the use of treatment efficacy as a driver of the tone of digital conversation. As noted in Table 1, a substantially lower percentage of conversations by Hispanics used treatment efficacy as a positive driver (19% vs. 28%, respectively) or the lack of treatment efficacy as a negative driver (9% vs. 23%, respectively). This rather suggests that the Hispanic population does not talk about depression treatment as often, likely reflecting the fear of being labeled as suffering from mental illness (a self-perceived stigma), except for the mention of their complications (26% vs. 20%, respectively). This complicated relationship of Hispanics with depression treatment is also reflected in the four stages of depression described by us based on the digital conversations.

As the attitude towards mental illness, including depression, can change over time, we used supervised NLP to classify the cross section of digital conversations into four stages of an individual’s experience with depression. These stages include digital conversation reflecting concern about the possibility of suffering from depression (Suspect stage), recently being diagnosed with depression (Diagnosed), and actively engaged in treatment (Treatment stage) or just living with it (Coping stage). Our analysis found that only slightly over half as many as conversations among Hispanics than non-Hispanics find them in the treatment phase of depression. In contrast, Hispanics are 1.8 times more frequently in the coping stage based on conversation analysis. Staying in the later stage means an awareness about having depression but dealing with it by any means other than clinical treatment. It may perhaps reflect the irrational fear of mental health treatment secondary to its stigma. The analysis of conversation topics even more clearly supports this lack of engagement with depression in a constructive way among Hispanics. Compared to non-Hispanics, only half as many Hispanics talk about therapies (25% vs. 12%, respectively) or deliberate the causes of depression (11% vs. 6%, respectively). In comparison, close to half (44%) of them discuss living with depression. The difference in topics discussed between the two populations can be noted in most of the stages of depression (Fig. 4). The infrequent topic of therapy in conversations by Hispanics is supported by the real-world data from the National Alliance on Mental Illness, which shows that each year approximately 33% of Hispanics with mental illness receive treatment compared to the average of 43% in the U.S [33]. A critical factor that should not be ignored when compared to non-Hispanics is the challenge related to immigration, availability of health insurance and eventual acculturation [8, 34, 35].

The analysis of attitude towards depression among Hispanics portrayed by these digital conversations demonstrates a similar lack of involvement with depression in them. We found a remarkable difference between non-Hispanics and Hispanics population regarding their active participation and involvement in managing depression. This difference is noted in the suspect (46% vs. 0%, respectively), diagnosed (35% vs. 9%, respectively), and the treatment (57% vs. 32%, respectively) phases. A clinically relevant finding is that Hispanics more frequently come to the diagnosis stage, from the suspect stage, with a resigned and hopeless attitude (67% vs. 44%), and maintain that attitude in the treatment phase as well. This mindset can potentially affect the patient–caregiver relationship, medication compliance, and, clinic adherence rates, all of which can create a vicious cycle of reaffirming their lack of engagement with, and benefit from treatment for depression. There is tremendous need to increase awareness about symptoms of depression and the benefits of therapy among Hispanics. Additionally, our results suggest that primary care and mental health providers need to be aware of a higher degree of a sense of hopelessness, and lack of belief in the effectiveness of treatment among Hispanics struggling with depression. Actively promoting their early and continued engagement with therapies for depression is required. Similarly, managing expectations by educating about expected delays of few weeks in emergence of benefits from anti-depressant may ameliorate some of the attitudes towards depression treatment. Previous research shows that Hispanics tend to view mental illness as a manifestation of weakness or a major flaw in their character and thus tend to reject the notion of having a mental disorder or needing psychiatric treatment [36, 37]. However, our research reveals that they possibly, in private, struggle with the thought of having depression.

The biggest strength of our research is that we were able to exploit the advances in AI tools like NLP to analyze digital conversation big data, which would not be possible otherwise. In addition, we used supervised machine learning whereby the judgement of clinicians about natural English language expressions and conversations was used to train the AI tools. Therefore, it means that the findings of our study can be applied on any English-speaking patient’s conversation during clinic visit by a care provider with a reasonable working knowledge of the language. A large dataset of close to half-million digital conversations provides definite robustness to our findings. An additional feature of our study is the advantage of analyzing public digital conversation on mental illness topics like depression. These conversations made outside of formal clinical or research environment and pooled from across the country help us to understand the mindset and attitudes towards depression from a previously unexplored vantage point. Along with the above strengths, our study has some limitations as well. Our findings only apply to the US population since only the conversations originating from IP addresses in the US that were not protected behind firewalls were used in the study. We only analyzed individuals whose ethnicity could be determined based on self-identification in their conversations or their public profile. Hispanics constitute 18.3% of US population [38], but they only contributed to 9.8% of all digital conversations analyzed by us. This could partly be due to the lower prevalence of depression in them compared to non-Hispanics population [39] or the fact that Hispanics are afraid to talk about their mental illness, and they will attempt multiple other avenues in fear of the stigma of accepting a psychiatric diagnosis. However, the role of analyzing only English language digital conversation cannot be ruled out. While a 2013 Pew research showed that 68% of Hispanics consider themselves proficient in English [40], it is possible we missed a small but a substantial portion of Hispanics posting online in Spanish, which we could not reliably analyze using NLP programs. Similarly, the limited availability of internet and technology in this community, which, however, is rapidly improving could be a reason as well [41]. Another limitation is that we did not collect gender- related data.

## Conclusion

Utilizing the digital conversations made by individuals during routine daily lives that are available in the public domain, our study reveals several key features of the Hispanic population’s relationship with depression. We found that they commonly have a negative, resigned, and hopeless attitude towards depression and lack active involvement with its management, which is in sharp contrast to non-Hispanics. This knowledge should particularly be used for formulating strategies to engage Hispanics communities in the awareness of depressive disorders. Based on our findings, we suggest possible communication strategies including the following key concepts: (1) Hispanic people suffering from depressive disorders are not weak, (2) there are biological factors associated with these mental illnesses, therefore they can be treated, (3) depressive episodes are not part of the normal transition in the development of individuals, (4) Hispanic suffering from depression do not have to “endure them resigned”, and (5) as with other racial groups, depressive disease in Hispanics can be effectively diagnosed and treated. Future studies validating our findings in clearly identified and characterized Hispanics are required to improve our patient’s mental well-being.

## Supplementary Information

**Additional file 1.**

## Data Availability

Not applicable.

## Abbreviations

AI: Artificial Intelligence
NLP: Natural Language Processing
IP: Internet Protocol
