# Supplementary material for: Digital conversations about depression among Hispanics and non-Hispanics in the US: a big‐data, machine learning analysis identifies specific characteristics of depression narratives in Hispanics
Source: Ann Gen Psychiatry. 2021 Nov 29;20:50. doi: 10.1186/s12991-021-00372-0 (PMC8630887; doi:10.1186/s12991-021-00372-0)
Supplement: Supplementary file 1 — Additional file 1. [file 12991_2021_372_MOESM1_ESM.pdf]

		Examples
**Depression journey**	Suspect	"I've been struggling with what I think is depression but sometimes I hate myself for thinking that and just think I'm being overdramatic".
	Diagnosed	"For better or worse having been diagnosed with depression and feeling like a complete failure for not being able to cope I have stated to read numerous books trying to discover why I might feel like I do and trying to find a way forward!"
	Treating	"I have been back on Lexapro 10mg for about three months now. I've noticed that I am CONTINUOUSLY hungry and am ALWAYS thinking about food. I never used to be like this and I hate it. I want to come off Lexapro because I know I'm slowly gaining weight and it's triggering my feelings of depression even more".
	Coping	"Everyday I live with a hole in my chest a emptiness that will never be filled, no matter where I am or who I'm with I'm never really happy. Ill find the worst in every situation and never think about the good. What's really been getting to me lately is how my depression will affect my relationships with those closest to me".
**Attitudes towards depression**	Struggling	"Everything feels more challenging when you're dealing with depression. Going to work, socializing with friends, or even just getting out of bed".
	Resigned/Hopeless	"I feel so alone and lonely. I feel like the world is beating on me. I've been crying for days, many sleepless nights and tiring days. How do I deal with depression? I already asked for help to some of my friends, but none of them is helpful".
	Involved	"Why should you be shameful for needing medication? It is NOT your fault that you are sick, and there is nothing wrong with being dependent on something, Everybody needs water, and some of us need meds - so what? I'm dependent on my SSRIs too and I don't see a problem with that. When they help me to get through the day, it is fine to take them".

Supplemtal Table 1: Examples of depression journey and attitudes towards depression.
